# Assessment of the Impact of Selected Industrial Wastewater on the Nitrification Process in Short-Term Tests

**DOI:** 10.3390/ijerph19053014

**Published:** 2022-03-04

**Authors:** Iwona B. Paśmionka, Janina Gospodarek

**Affiliations:** Department of Microbiology and Biomonitoring, Faculty of Agriculture and Economics, University of Agriculture in Krakow, Mickiewicza 24/28 Av., 30059 Krakow, Poland; janina.gospodarek@urk.edu.pl

**Keywords:** nitrifying bacteria, nitrification inhibition, chemical wastewater, activated sludge

## Abstract

Many chemical compounds can inhibit the nitrification process, especially organic compounds used in the chemical industry. This results in a decrease in the nitrification intensity or even a complete termination of this process. As the technological design of the selected municipal and industrial wastewater treatment plant (WWTP) assumed the dephosphation process, without taking into account nitrification, it was necessary to reduce the concentration of ammonium nitrogen in the treated sewage supplied to the Vistula River. Therefore, the aim of the research was to determine the inhibition of nitrification in the activated sludge method under the influence of industrial wastewater from the production of various organic compounds and to select the most toxic wastewater in relation to nitrifiers. The assessment of nitrification inhibition was carried out on the basis of the method of short-term (4-h) impact of the tested sewage on nitrifying bacteria in the activated sludge. The research covered nine different types of chemical sewage, including wastewater from the production of synthetic rubbers, styrene plastics, adhesives, solvents and emulsifiers. The nitrification process was inhibited to the highest degree by wastewater from the production of styrene-butadiene rubbers (72%). Only wastewater from the production of methacrylate (polymethyl methacrylate) had the lowest degree of inhibition: 16%. These wastewaters also have a toxic effect on the entire biocenosis and adversely affect the structure of activated sludge flocs. The attempts to filter toxic wastewater through the ash basins significantly relieved the inhibition of nitrification.

## 1. Introduction

The development of the chemical industry is one of the main factors of civilization progress; at the same time, it has the largest share in the degradation of the natural environment. The full involvement of industry in environmental protection requires large financial outlays related to the need to develop various methods of industrial wastewater treatment. Wastewater pre-treated at production departments is usually directed to an industrial wastewater treatment plant. In organic chemistry plants it is usually a mechanical and biological WWTP. It is well known that an important issue is the removal of biogenic compounds from wastewater, i.e., nitrogen compounds, because after their introduction into the environment they can cause negative effects, mainly associated with eutrophication of surface waters, but also with groundwater pollution [1,2]. Moreover, the presence of excessive concentrations of nitrogen compounds in aquatic environments negatively affects public health. The need to eliminate these compounds from wastewater is imposed by applicable legal regulations, which are increasingly tightening, including allowable concentrations of nitrogen in wastewater discharged into receivers.

The removal of nitrogen compounds from wastewater is primarily carried out by biological methods using nitrification and denitrification processes [3]. Microorganisms participating in these processes are sensitive to various environmental factors, which include, among others: temperature, pH, concentration of dissolved oxygen, age of activated sludge, concentration of NH_4_-N, and the presence of inhibiting substances [4]. Nitrifying bacteria are more sensitive to toxic substances contained in wastewater than other bacteria involved in the wastewater treatment process. Nitrification is inhibited by many substances, both organic and inorganic. Especially dangerous are those that come from the chemical industry. These include certain metals, organic sulphur compounds, aniline derivatives, phenols, cyanides and many more. The mechanism of nitrification inhibition may vary. It may consist of delivering an additional proton inside the cell. This disrupts the pH gradient on both sides of the cell membrane, which in turn prevents ATP (adenosine triphosphate) synthesis. Another mechanism is that the inhibitor reacts with nitrite oxidoreductase, which deactivates this enzyme and blocks nitrate oxidation. [5,6].

The increased requirements for the quality of treated wastewater contribute to the development of advanced biogen removal technologies, which are an alternative to the expensive expansion of existing biological systems. In the case of biological nitrogen removal from wastewater, these trends justify the increased interest in the processes reducing the nitrogen load on the main process line [7,8].

At the end of the last century, the Chemical Company Dwory S.A. in Oświęcim (Poland) began construction of a post-production WWTP. At the same time, the Oświęcim City Council was planning to build an independent municipal wastewater treatment plant. Therefore, it was decided to specify the requirements and possibilities of joint treatment of industrial and municipal sewage in one treatment plant, using the already implemented part of the investment for industrial sewage. In connection with the above, long-term research was started to check whether urban and industrial wastewater can be treated jointly, in accordance with the proposed technology. The experiments carried out by the WWTP employees at that time showed no toxicity of mixed wastewater, and the sludge did not show changes in the structure and composition of biocenosis (unpublished studies). Thus, the intended purification results were expected.

Shortly after the facility was commissioned, it turned out that the intensive production and large diversity of organic compounds produced by the Chemical Company, which in the meantime changed its name to Synthos S.A., hinders the work of the treatment plant. Synthos S.A. is a chemical concern whose activity consists of the production and delivery of solutions for the needs of various industries. The company produces synthetic rubbers, styrene plastics, plant protection chemicals as well as dispersions and latexes, whose recipients are companies from all over the world. Uncontrolled discharges of post-production sewage into the industrial sewage system have started to cause emergency states, exceeding the tolerance limits by microorganisms. There were periods of complete disappearance or reduction of the nitrification process efficiency in the biological treatment system, which was manifested by an increase in the concentration of NH_4_-N in treated wastewater discharged into the Vistula River. Therefore, the need for stable nitrification has become a fundamental problem of the treatment plant. Despite meeting the technological requirements, this task turned out to be extremely difficult, because the nitrification process in the discussed system is limited by the presence of inhibiting substances contained in industrial wastewater flowing from the Synthos S.A. production plants.

The need to comply with already introduced requirements and the prospect of further tightening of regulations forces us to look for the possibility of intensifying biological nitrogen removal. Therefore, the aim of the study was to assess the inhibitory impact of industrial wastewater from the production of various organic compounds at Synthos S.A. on the nitrification process in activated sludge, microorganisms’ activity, biocenosis and the structure of activated sludge flocs at the Municipal and Industrial Wastewater Treatment Plant in Oświęcim (Poland).

## 2. Materials and Methods

The WWTP in Oświęcim is one of the largest municipal industrial wastewater treatment plants in Poland. It is located in south-eastern Lesser Poland (50°02′17.1″ N 19°19′13.8″ E) and receives municipal sewage from the city and commune of Oświęcim, as well as industrial sewage from the Chemical Company Synthos S.A.

**Figure 1:**
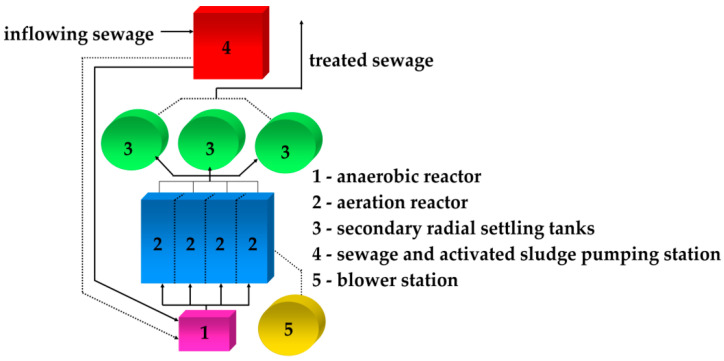
Diagram of the biological sewage treatment system in the Municipal and Industrial Wastewater Treatment Plant in Oświęcim, Poland.

Biological treatment is carried out based on the technology of low load activated sludge. The biological system consists of an anaerobic chamber, into which industrial and municipal sewage mixed in a 2:1 ratio is introduced from four aeration chambers, equipped with mixers and a system for fine bubble compressed air aeration from three secondary radial settling tanks, blower station and pumping station of activated sludge (Figure 1).

The research was conducted from March to October 2021. The nine selected wastewaters were analysed in triplicate. In order to determine the inhibition of nitrification, studies in model systems were undertaken in activated sludge. The research covered industrial wastewater from the production of styrene butadiene rubbers, acrylonitrile rubbers, emulsifiers, polyvinyl acetate, polyvinyl chloride, styrene, terpenes, solvents: ethyl acetate, butyl acetate and methacrylate. The chemical composition of the tested wastewater is presented in Table 1.

The assessment of the inhibitory effect of post-production wastewater on the nitrification process was carried out based on the International Standard ISO/DIS-9509, which specifies the method for assessing the short-term inhibitory effect of the tested wastewater on nitrifying bacteria present in activated sludge [8,9,10].

**Figure 2:**
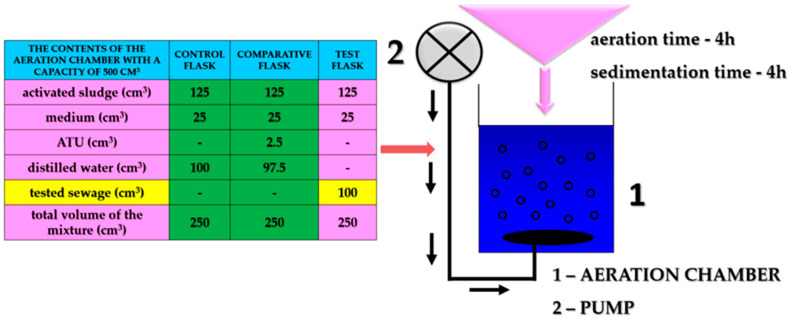
Assessment of the inhibitory effect of industrial wastewater on the nitrification process–experiment scheme.

In order to carry out the tests, a properly prepared activated sludge was introduced into the conical flasks. Then, medium based on ammonium sulphate and sodium bicarbonate was added to each flask. The control sample was a mixture of activated sludge with distilled water and the medium. The reference sample was a mixture of activated sludge with medium, comparative inhibitor–allylthiourea (ATU) and distilled water. Test sewage was added to the remaining conical flasks. The systems prepared in this way were aerated for 4 h with moist, compressed air. The diagram of the conducted experiment and technological parameters of the activated sludge used for the research are presented in Figure 2 and Table 2.

After 4 h of aeration, a sample was taken from each flask, in which, after filtering, the concentration of NH_4_-N and NO_3_-N was determined by colorimetry. The determinations were made in triplicate. The test results are presented as the percentage of nitrification inhibition calculated according to Equation (1) [9]:

(1) $$\%\mathrm{IN}=\frac{C_{C}\;-\;C_{T}}{C_{C}\;-\;C_{B}}\cdot 100$$

C_C_–average concentration of oxidized forms of nitrogen in the control flask after 4 h of aeration, mg·dm^3^,

C_T_–average concentration of oxidized forms of nitrogen in the flask with the tested sewage after 4 h of aeration, mg·dm^3^,

C_B_–average concentration of oxidized forms of nitrogen in a flask with a comparative inhibitor (ATU) after 4 h of aeration, mg·dm^3^.

The results were statistically analysed using Statistica 13.1 (StatSoft, Inc., Tulsa, OK, USA). The analysis of variance (ANOVA) was calculated and the significance of differences between means was verified by Tukey’s test (α = 0.05). In order to test the strength of the relationship between %IN and COD (chemical oxygen demand) in the analysed wastewater, a simple correlation was used to test the relationship between two features (x, y). The following scale was adopted for the interpretation of the obtained results:

r_xy_ = 0: variables are not correlated

0 < r_xy_ < 0.2: no linear dependence

0.2 ≤ r_xy_ < 0.4: weak dependence

0.4 ≤ r_xy_ < 0.7: moderate dependence

0.7 ≤ r_xy_ < 0.9: quite strong dependence

0.9 ≤ r_xy_ < 1: very strong dependence

## 3. Results and Discussion

An important factor limiting or even preventing the nitrification process in the biological system are the inhibitors inflowing along with industrial wastewater [2,6,11,12,13,14,15,16,17]. In connection with the above, research on inhibition of the nitrification process was started in order to determine the most toxic wastewater stream. The obtained results allowed the selection of particularly dangerous streams of wastewater from the production of various organic compounds, which have a detrimental effect on the treatment process due to:

(1) Nitrification inhibition;
(2) Changes in the structure of activated sludge flocs;
(3) Toxic effect on activated sludge microorganisms.

**Figure 3:**
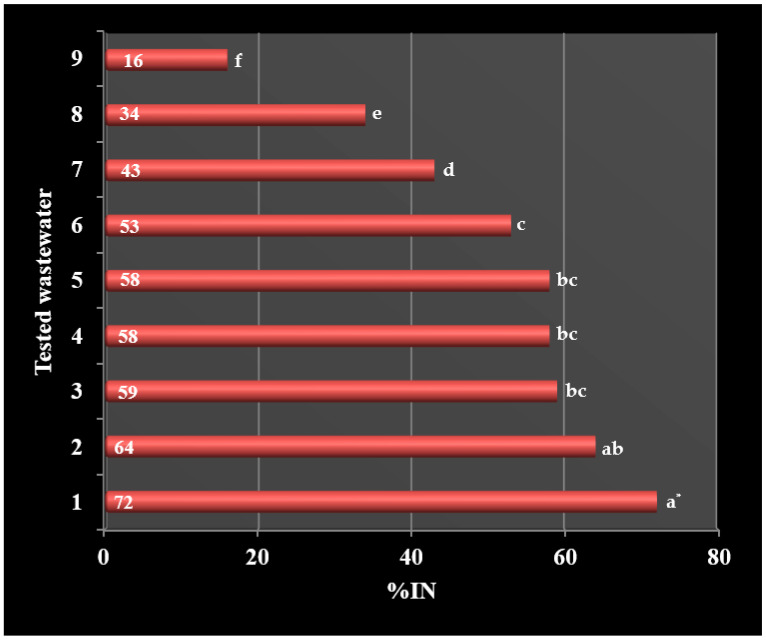
Percentage inhibition of nitrification. 1 styrene butadiene rubbers; 2 solvents: butyl acetate, ethyl acetate; 3 styrene; 4 acrylonitrile rubbers; 5 terpenes; 6 emulsifiers; 7 polyvinyl acetate; 8 polyvinyl chloride; 9 methacrylate. * Averages marked with the same letters are not significantly different by Tukey’s test (α = 0.05).

The nitrification process was inhibited to the highest degree by wastewater from the production of styrene butadiene rubbers (72%), solvents (64%), styrene (59%), acrylonitrile rubbers (58%) and terpenes (58%). Wastewater from the production of emulsifiers (53%), polyvinyl acetate (43%) and polyvinyl chloride (34%) was characterized by a high degree of inhibition (Table 3, Figure 3).

**Figure 4:**
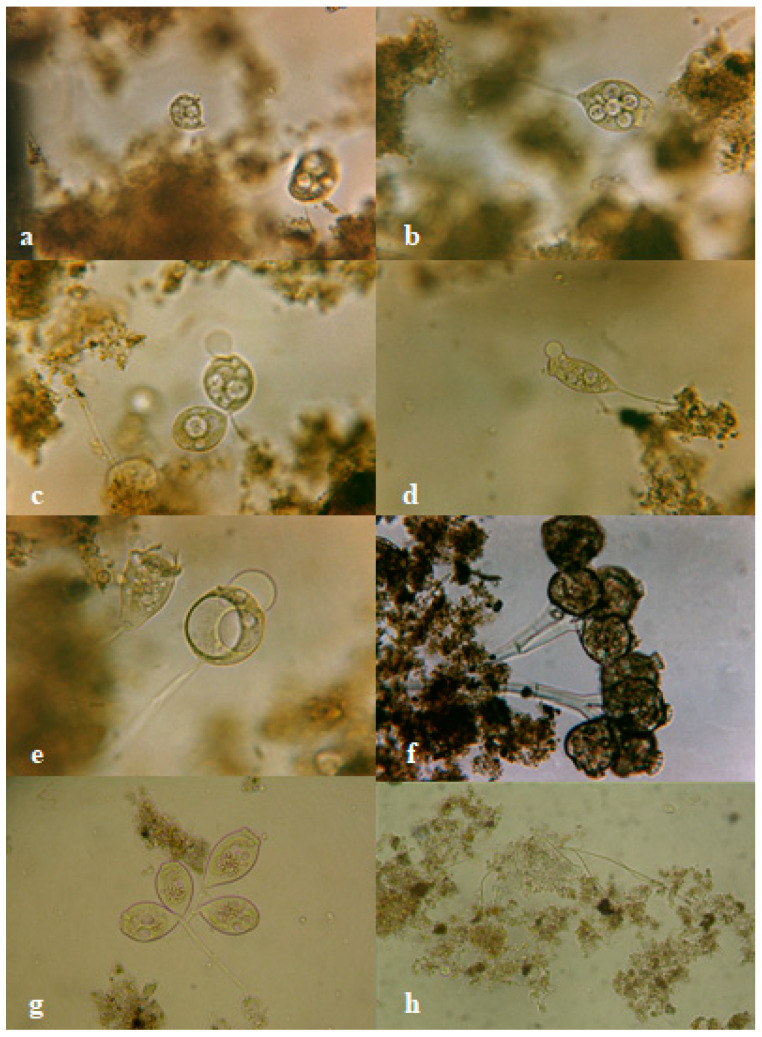
*Aspidisca costata* (**a**), *Vorticella communis* (**b**), *Vorticella microstoma* (**c**), *Vorticella elongata* (**d**), *Vorticella convallaria* (**e**), *Epistylis* spp. (**f**), *Carchesium polypinum* (**g**) damaged due to sewage from the production of acrylonitrile rubbers and activated sludge flocs (**h**) with visible perforations and holes (photo by Iwona B. Paśmionka).

These wastewaters are potential carriers of numerous nitrification inhibitors, of which the most important are versenic acid, tricresol, acrylic acid, acrylonitrile, acetonitrile, tertiary dodecyl mercaptan and ethylene glycol [1,18]. It should be noted that wastewater from the production of acrylonitrile rubbers, despite a slightly lower degree of inhibition, shows a very toxic nature. These compounds are produced periodically, but the sewage generated significantly slows down nitrification, and in the biological system over time causes complete inhibition of this process, which is confirmed by literature data [19,20]. In addition, they have a negative impact on protozoa occurring in activated sludge, because they increase the number of dead or damaged ciliates, especially crawling from the genus *Aspidisca* (Figure 4a) and sedentary from the genus *Vorticella* (Figure 4b–e), *Epistilis* (Figure 4f) and *Carchesium* (Figure 4g). In activated sludge flocs they cause numerous perforations (Figure 4h).

Probably the factor determining the toxicity of these wastewaters is the time of interaction of the inhibitor with the activated sludge, which negatively affects both the nitrification process, the state of biocenosis and the structure of activated sludge flocs. This phenomenon is justified in the literature. Acrylic acid present in these wastewaters at a concentration > 10.0 mg·dm^−3^ completely inhibits the nitrification process, and in an amount above 100.0 mg·dm^−3^ is toxic to protozoa [12,21]. Another very strong nitrification inhibitor that appears in wastewater during the production of acrylonitrile rubbers is tertiary dodecyl mercaptan, which, already at a concentration of 3.0 mg·dm^−3^, inhibits nitrification by 75%, as well as sodium thiuram, which also has a high degree of inhibition. In addition, acetonitrile is also a toxic substance, which has a negative effect on activated sludge [22,23].

A relatively high degree of nitrification inhibition is characterized by wastewater from the production of solvents: polyvinyl acetate and polyvinyl chloride (Table 3, Figure 3). These wastewaters contain, among others, polyvinyl alcohol, methanol, butanol and ethanol, among which the strongest inhibiting properties has ethyl alcohol, which already, at a concentration of 2.4 mg·dm^−3^, inhibits the nitrification process [22,24].

Only wastewater from the production of methacrylate (polymethyl methacrylate) had the lowest degree of inhibition, 16% (Table 3, Figure 3). This phenomenon is not confirmed in the literature, because at least two organic compounds used in the production of polymethyl methacrylate are strong inhibitors of the nitrification process [22]. These compounds include acrylic acid, which at a concentration of just above 10.0 mg·dm^−3^ inactivates the nitrification process, as well as methacrylamide, which has toxic effects at a concentration lower than 25.0 mg·dm^−3^. Perhaps the above-mentioned compounds do not enter the industrial sewage system or are diluted with wash water, which reduces the inhibition of nitrification.

**Figure 5:**
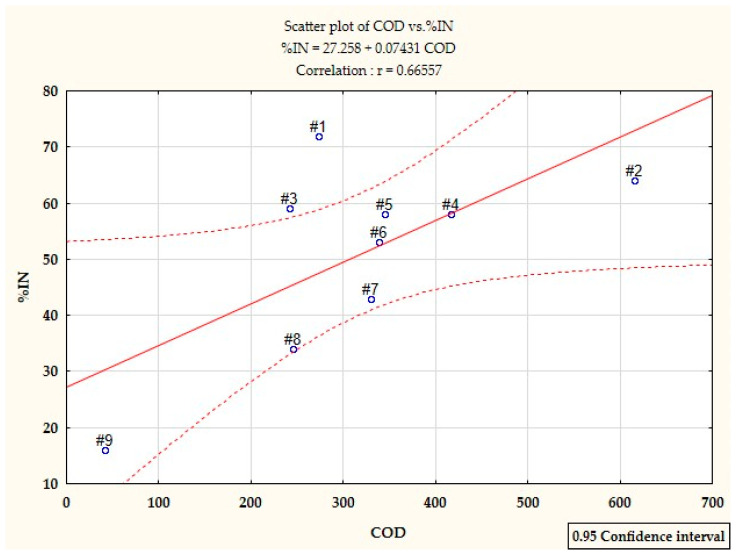
Scatter plot showing the strength of the correlation between% IN and COD of the analysed wastewater: #1 styrene butadiene rubbers; #2 solvents: butyl acetate, ethyl acetate; #3 styrene; #4 acrylonitrile rubbers; #5 terpenes; #6 emulsifiers; #7 polyvinyl acetate; #8 polyvinyl chloride; #9 methacrylate.

The statistical analysis showed significant differences in the inhibition of nitrification between wastewater from the production of styrene butadiene rubbers and other wastewater (Tukey’s test; *p* < 0.05), except for wastewater from the production of solvents butyl acetate and ethyl acetate. On the other hand, the inhibition of nitrification under the influence of wastewater directly from the production of styrene, acrylonitrile rubbers and terpenes did not differ significantly (Tukey’s test; *p* > 0.05), (Figure 3). The conducted correlation analysis showed a rectilinear relationship between %IN and COD of the tested wastewater. The correlation coefficient r_xy_ = 0.66557 indicates a moderate dependence of %IN on COD. According to the scatterplot, the greatest strength of correlation is shown by the wastewater from the production of acrylonitrile rubbers, terpenes and emulsifiers (Figure 5).

**Figure 6:**
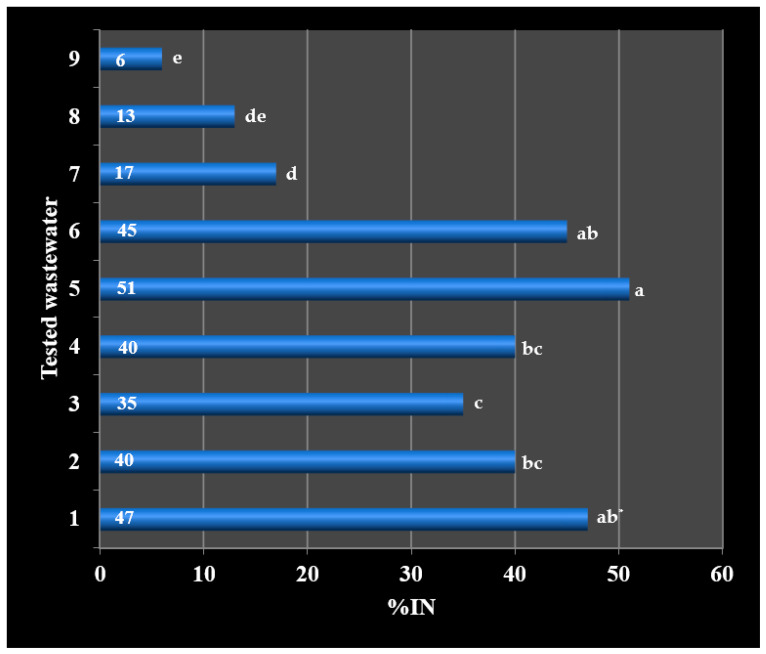
Influence of the filtering process on the percentage of nitrification inhibition. 1 styrene butadiene rubbers; 2 solvents: butyl acetate, ethyl acetate; 3 styrene; 4 acrylonitrile rubbers; 5 terpenes; 6 emulsifiers; 7 polyvinyl acetate; 8 polyvinyl chloride; 9 methacrylate. * Averages marked with the same letters are not significantly different by Tukey’s test (α = 0.05).

Due to the unfavourable results that were obtained in the first series of tests, it was decided to check whether it is possible to reduce the inhibitory effect of post-production sewage on the nitrification process. In connection with the above, the tested wastewater was subjected to filtration in an ash basin. The results obtained in the samples taken at the outlet of the ash basins indicate that the inhibitory effect is largely reduced during sewage filtration on ash (Table 4, Figure 6).

The exception was sewage from the production of terpenes and emulsifiers, whose negative impact on the nitrification process slightly decreased. The inhibitory effect of wastewater from the production of styrene butadiene rubbers has decreased from 72% to 47%. In the case of wastewater from solvent production, the filtration process has contributed to the reduction in nitrification inhibition from 64% to 40% (Figure 3 and Figure 6).

**Figure 7:**
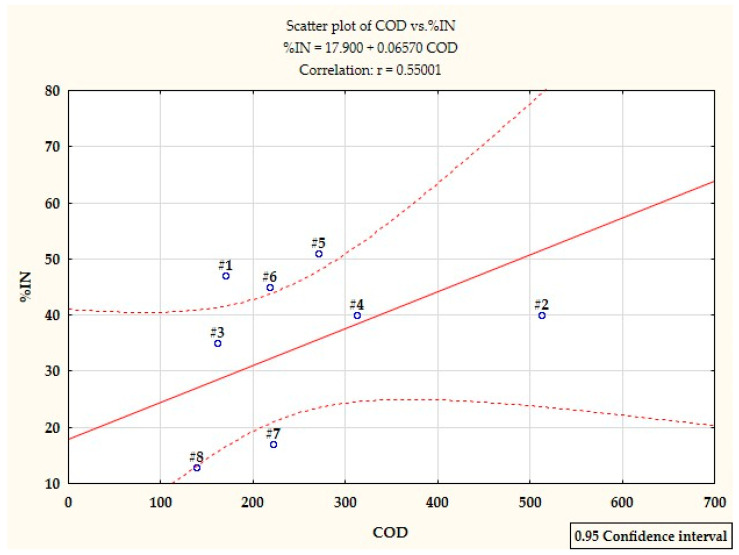
Scatter plot showing the strength of the correlation between% IN and COD of wastewater filtered in ash basins: #1 styrene butadiene rubbers; #2 solvents: butyl acetate, ethyl acetate; #3 styrene; #4 acrylonitrile rubbers; #5 terpenes; #6 emulsifiers; #7 polyvinyl acetate; #8 polyvinyl chloride.

After the filtration process of wastewater in ash basins, statistical analysis showed the highest significant differences in nitrification inhibition between wastewater from the production of styrene butadiene rubbers and wastewater from the production of polyvinyl acetate, polyvinyl chloride and methacrylate. In addition, it was found that the samples of filtered sewage from the production of styrene, acrylonitrile rubbers and terpenes differ significantly from unfiltered sewage (Tukey’s test; *p* < 0.05), (Figure 3 and Figure 6). The obtained results suggest a statistically significant reduction in the effect of nitrification inhibition under the influence of the filtration process (Tukey’s test; *p* < 0.05), except for wastewater from the production of terpenes and emulsifiers. The process of filtering wastewater from the production of polyvinyl acetate, polyvinyl chloride and methacrylate increased the efficiency of the nitrification process by approx. 60%. The filtration process had the slightest effect on the toxicity of wastewater from the production of terpenes and emulsifiers, the nitrification efficiency increased by only 12 and 15% (Tukey’s test; *p* > 0.05), (Table 5). An additional correlation analysis showed a rectilinear relationship between %IN and COD, similarly to unfiltered sewage. The correlation coefficient r_xy_ = 0.55001 also indicates a moderate dependence of %IN on the COD of sewage filtered in ash basins. According to the scatter diagram, the highest correlation strength is shown in the wastewater from the production of styrene and acrylonitrile rubbers (Figure 7). As %IN turned out to be statistically different for all the tested sewage samples, it can be assumed that the inhibition of nitrification at the Municipal and Industrial Wastewater Treatment Plant in Oświęcim is caused by the presence of toxic substances in the sewage from Synthos S.A.

Nitrifying bacteria are sensitive to numerous toxic substances and inhibitory agents. Many chemical compounds have not yet been tested for their toxicity against nitrifiers [7,25]. In addition, in an environment such as wastewater, especially wastewater from the chemical industry, many compounds interact with each other, creating new, sometimes more toxic connections [26,27]. The potential reaction of nitrifying bacteria found in activated sludge to inhibitory substances is acute or chronic stress [3,5]. Acute stress usually leads to an immediate decrease in the intensity of the nitrification process or its complete interruption, which was observed in the conducted research (Table 3). Restoration of stable nitrification depends on many external factors such as temperature, pH, age of activated sludge or elimination of inhibiting compounds from the system [4,28]. Chronic stress can often occur without visible signs and can only be noticed by comparing the nitrification activity of the tested sludge with the nitrification activity of the sludge in other systems. In this situation, the rate of nitrification is usually lower and may require the use of various means to achieve the required degree of ammonia nitrogen oxidation [29,30]. One of the possibilities to increase the efficiency of the nitrification process is to filter toxic wastewater before entering it into the biological treatment system (Table 4 and Table 5).

## 4. Conclusions

1. Industrial wastewater from the chemical company Synthos S.A. contain numerous toxic substances that significantly inhibit the nitrification process.
2. The most dangerous industrial streams include wastewater from the production of styrene butadiene rubbers, solvents (butyl acetate, ethyl acetate), styrene, acrylonitrile rubbers, terpenes and emulsifiers, which inhibit nitrification above 50%.
3. To a slightly lower degree, the nitrification process is inhibited by wastewater from the production of polyvinyl acetate (43%) and polyvinyl chloride (33%).
4. Wastewater from methacrylate production turned out to be the least toxic to nitrifying bacteria and showed the lowest degree of nitrification inhibition (16%).
5. Wastewater from the production of styrene-butadiene and acrylonitrile rubbers, filtered in ash basins, show a much lower degree of nitrification inhibition than the same wastewater directed to the sewage treatment plant without the filtration process.
6. The wastewater filtering process in ash basins significantly reduces nitrification inhibition, even about 60% for some wastewater.
7. Under static testing, wastewater from the production of styrene butadiene rubbers has a much higher degree of nitrification inhibition than the wastewater from the production of acrylonitrile rubbers. This phenomenon is not confirmed in the biological purification system, because as the production time of acrylonitrile rubbers increases, the efficiency of the nitrification process is significantly reduced or even completely ceases, which proves that a long time of exposure of the toxic factor enhances the inhibition of nitrification. This is also confirmed by the analysis of the correlation between %IN and COD of these wastewater, both before and after filtration.
8. The conducted research allowed to select the most toxic industrial wastewater. The performed analyses suggest that the main cause of the inhibition of nitrification are the toxins contained in the sewage.
9. The obtained results indicate the need for further research to determine the inhibitory concentration (IC) of the nitrification process for the most toxic wastewater from the production of styrene butadiene rubbers, solvents: butyl acetate, ethyl acetate, styrene, acrylonitrile rubbers and terpenes.

## Figures and Tables

**Table 1:** Chemical composition of the analysed wastewater.

Sewage from Production	Chemical Compounds of Sewage
styrene butadiene rubbers	latex, silicone emulsion, technical olein, butadiene, styrene, versenic acid, alkyl benzene sulfonic acid, acetic acid, butylcatechol, tricresol
solvents: butyl acetate, ethyl acetate	butanol, acetic acid, acetaldehyde, ethanol, carbide
styrene	ethylbenzene, p-tert-butylcatechol
acrylonitrile rubbers	acrylic acid, ethyl glycol, sodium acetate, butadiene, acrylonitrile, acetonitrile, stearin, versenic acid, tertiary dodecyl mercaptan, thiuram sodium
terpenes	balsamic turpentine
emulsifiers	balsamic rosin, balsamic turpentine
polyvinyl acetate	distilled vinyl acetate, polyvinyl alcohol, crude carbide, dibutyl phthalate, rectified methanol, lauroyl peroxide
polyvinyl chloride	distilled vinyl chloride
methacrylate	methyl methacrylate, alpha-azodi-isobutyronitrile, dioctyl phthalate, technical stearin, butanol, acrylic acid, methacrylamide, butyl acrylate, citric acid, distilled vinyl acetate, butyl methacrylate, toluene, benzoyl peroxide, ethyl acetate

**Table 2:** Technological parameters of the activated sludge used for the research.

Parameter	Unit	Value
temperature	°C	14.9
oxygenation	mg O_2_·dm^−^^3^	1–3
aeration time	h	4–5
total suspension of the activated sludge in the aeration chamber	g·dm^−^^3^	3.5–5.0
total suspension in excess sludge	g·dm^−^^3^	6.0–8.0
excess sludge increase	m^3^·d^−1^	300–400
recirculation	%	120
dry mass of activated sludge	%	1.64
the age of the activated sludge	days	13–14
BOD_5_	kgBOD_5_·kg_d.m._·d^−1^	0.11–0.19

**Table 3:** Influence of industrial wastewater on the nitrification inhibition process in activated sludge.

Sample	pH	COD (mg O_2_·dm^−3^)	Average Concentration NH_4_-N (mg·dm^−3^)		Average Concentration NO_3_-N (mg·dm^−3^)	
			Before Incubation	After 4 h of Incubation	Before Incubation	After 4 h of Incubation
C	7.4	-	56.0	24.3	0.0	19.2
ATU	7.4	-	56.0	36.2	0.0	0.1
1	7.8	272.7	56.0	39.7	0.0	5.4
2	7.5	615.5	56.0	27.9	0.0	7.0
3	7.4	242.1	56.0	32.0	0.0	8.0
4	7.0	416.5	56.0	35.2	0.0	8.2
5	7.5	345.6	56.0	26.9	0.0	8.2
6	7.6	339.0	56.0	45.4	0.0	9.1
7	7.7	329.8	56.0	14.0	0.0	10.9
8	7.3	245.6	56.0	21.4	0.0	12.8
9	7.3	41.9	56.0	18.0	0.0	16.1

C–control; ATU–comparative inhibitor; production of: 1 styrene butadiene rubbers; 2 solvents: butyl acetate, ethyl acetate; 3 styrene; 4 acrylonitrile rubbers; 5 terpenes; 6 emulsifiers; 7 polyvinyl acetate; 8 polyvinyl chloride; 9 methacrylate. MLVSS—3.571 g·dm^−3^, specific nitrification rate—0.83, nitrifying activity of activated sludge—1.33

**Table 4:** Impact of the filtration process on the degree of nitrification inhibition in activated sludge.

Sample	pH	COD (mg O_2_·dm^−3^)	Average Concentration NH_4_-N (mg·dm^−3^)		Average Concentration NO_3_-N (mg·dm^−3^)	
			Before Incubation	After 4 h of Incubation	Before Incubation	After 4 h of Incubation
C	7.6	-	56.0	21.4	0.0	22.7
ATU	7.6	-	56.0	39.5	0.0	0.1
1	7.3	170.0	56.0	28.5	0.0	12.0
2	7.6	512.7	56.0	18.6	0.0	13.6
3	7.2	161.8	56.0	25.7	0.0	14.7
4	7.1	312.6	56.0	29.1	0.0	13.6
5	7.4	271.3	56.0	19.6	0.0	11.2
6	7.1	218.0	56.00	32.6	0.0	12.6
7	7.0	221.4	56.0	10.3	0.0	18.8
8	7.2	138.5	56.0	13.3	0.0	19.7
9	7.4	16.4	56.0	11.4	0.0	21.4

C–control; ATU–comparative inhibitor; production of: 1 styrene butadiene rubbers; 2 solvents: butyl acetate, ethyl acetate; 3 styrene; 4 acrylonitrile rubbers; 5 terpenes; 6 emulsifiers; 7 polyvinyl acetate; 8 polyvinyl chloride; 9 methacrylate. MLVSS—3.755 g·dm^−3^, specific nitrification rate—1.20, nitrifying activity of activated sludge—1.50.

**Table 5:** Reduction of the effect of nitrification inhibition under the influence of sewage filtration in ash basins.

Sample ^1^	%IN		Increase in the Efficiency of the Nitrification Process (%)
	Before Filtration	After Filtration	
1	72 a *	47 b	35
2	64 a	40 b	37
3	59 a	35 b	41
4	58 a	40 b	31
5	58 a	51 a	12
6	53 a	45 a	15
7	43 a	17 b	60
8	34 a	13 b	61
9	16 a	6 b	62

^1^ production of: 1 styrene butadiene rubbers; 2 solvents: butyl acetate, ethyl acetate; 3 styrene; 4 acrylonitrile rubbers; 5 terpenes; 6 emulsifiers; 7 polyvinyl acetate; 8 polyvinyl chloride; 9 methacrylate. * Averages marked with the same letters are not significantly different by Tukey’s test (α = 0.05).

## Data Availability

Not applicable.
